# Gastrointestinal point-of-care ultrasound for enteral nutrition management in critically ill adults: a scoping review

**DOI:** 10.3389/fnut.2026.1972620

**Published:** 2026-10-12

**Authors:** Junjie Hua, Wang Ju, Qiuying Gu, Jun Wu, Weiqing Zhang

**Affiliations:** 1 Shanghai Jiao Tong University School of Nursing, Shanghai, China; 2 Department of Cardiac Critical Care Medicine, Ruijin Hospital, Shanghai Jiao Tong University School of Medicine, Shanghai, China; 3 Department of Critical Care Medicine, Ruijin Hospital, Shanghai Jiao Tong University School of Medicine, Shanghai, China

**Keywords:** critical illness, enteral nutrition, gastrointestinal tract, intolerance, point-of-care ultrasound

## Abstract

**Background:**

Gastrointestinal point-of-care ultrasound has been investigated as a non-invasive, bedside adjunct for gastrointestinal assessment and enteral-feeding decision-making in critical care; however, existing protocols and their outcome reporting remain fragmented. This scoping review mapped the existing evidence landscape on the use of gastrointestinal point-of-care ultrasound in the management of enteral nutrition in critically ill adults.

**Methods:**

Comprehensive searches in 10 databases (PubMed, EMBASE, Web of Science, Cochrane, CINAHL, Scopus, China National Knowledge Infrastructure, Wanfang, VIP, China Biological Medicine Literature Database) identified 5,123 records. Studies focusing on ultrasound-assessed gastrointestinal function to guide enteral nutrition, or to predict enteral nutrition intolerance and clinical prognosis, were screened, summarized, and descriptively analyzed.

**Results:**

49 unique studies were identified, including 36 interventional and 13 observational studies. Geographically, the synthesized evidence was heavily concentrated in China. Ultrasound indicators for gastrointestinal function included the gastric antrum (gastric residual volume, motility index, cross-sectional area), colon (colon diameter and peristaltic frequency), small intestine (small intestine diameter, wall thickness, peristalsis), and superior mesenteric artery hemodynamics. Observational studies showed Area Under the Curve point estimates frequently around or above 0.70, with substantial heterogeneity and no independent external validation reported for enteral nutrition intolerance and mortality. Among interventional studies, time to achieve feeding goals and intensive care unit length of stay were the most frequently evaluated outcomes (27/36), whereas in-hospital mortality was evaluated in only 8 of 36 studies; outcome reporting was heterogeneous and is presented descriptively.

**Conclusion:**

The synthesized literature suggests the potential utility of gastrointestinal point-of-care ultrasound as an objective, non-invasive bedside tool for gastrointestinal assessment and feeding protocol modulation. However, because no formal critical appraisal or pooled comparative or diagnostic analysis was performed, this review cannot establish clinical effectiveness, universal thresholds, or patient-important benefit. Future research priorities include consensus definitions and acquisition standards, effect estimates with confidence intervals and patient-important outcomes, inter-operator reliability and training evaluation, and external validation across diverse health systems.

## 1 Introduction

Critically ill patients undergo a hypercatabolic and hypermetabolic state driven by their underlying pathologies, leading to accelerated nutrient breakdown, such as protein degradation, and subsequent negative nitrogen balance (1). For patients unable to tolerate oral feeding, enteral nutrition (EN) not only provides essential substrates to sustain organ function but also mitigates disease severity, reduces complication rates (2), shortens hospital length of stay (3), and improves patient prognosis (4). However, due to severe physiological dysregulation, critically ill patients exhibit a high susceptibility to enteral nutrition intolerance (ENI), which significantly compromises the therapeutic efficacy of enteral feeding strategies (5). No universally accepted definition of enteral feeding intolerance exists in the critical care literature. The 2012 ESICM Working Group proposed inability to deliver at least 20 kcal/kg body weight/day enterally within 72 h of a feeding attempt as one operational criterion (6), whereas subsequent work has emphasized a pragmatic, treatment-based definition and the heterogeneity of the associated gastrointestinal signs (7). In this review, ENI was accepted as defined by each primary study, and the component criteria (gastrointestinal symptoms, gastric residual volume thresholds, and caloric-delivery criteria) were charted rather than harmonized retrospectively. In the context of critical care, the development of ENI or the failure to meet nutritional targets significantly increases the risk of infection (8), elevates the incidence of multiple organ dysfunction syndrome, and profoundly impacts disease-related mortality (9).

The primary objective of EN management in the ICU is to optimize nutrient delivery while proactively preventing the onset of ENI. Currently, strategies to guide EN and mitigate intolerance rely heavily on monitoring Gastric Residual Volume (GRV) and assessing the presence and severity of gastrointestinal symptoms (6, 7). Clinicians utilize these parameters to titrate feeding rates or determine the necessity for feeding interruption. However, current management strategies face significant limitations: traditional GRV measurements lack diagnostic accuracy (10), and clinical signs are often subjective and non-specific. Crucially, these assessments are inherently reactive rather than predictive; they typically identify dysfunction only after overt clinical manifestations, such as vomiting or abdominal distension, have occurred. Consequently, this approach often fails to detect latent gastrointestinal dysfunction in a timely manner, resulting in the development of ENI and the subsequent impediment of essential nutritional therapy.

Given these diagnostic challenges, there is a critical need for a more objective, real-time tool to continuously monitor gastrointestinal function during EN therapy. Point-of-care ultrasound (POCUS) has emerged as a promising solution, offering a non-invasive, bedside approach for direct anatomical and functional assessment. Its capacity for real-time visualization facilitates rapid clinical decision-making and allows for the dynamic tracking of gastrointestinal physiological changes, directly addressing the “lag” inherent in symptom-based assessments (11).

Recent evidence syntheses have addressed selected components of GI POCUS. Specifically, a recent systematic review and meta-analysis quantified the diagnostic accuracy of gastrointestinal ultrasound for predicting feeding intolerance (12), a narrative review proposed a structural gastrointestinal ultrasound protocol (13), and a recent scoping review mapped bedside ultrasound for gastrointestinal function and enteral-nutrition guidance (14). Building on these studies, the present review updates the search through June 18, 2026 and jointly maps interventional feeding-titration algorithms, predictive cohorts, multi-segmental parameters, and outcome-reporting patterns across English- and Chinese-language literature.

However, to our knowledge, no published synthesis has yet jointly integrated (1) anatomical and hemodynamic parameter distributions, (2) interventional feeding titration algorithms, (3) multi-segmental predictive parameters, and (4) clinical outcome reporting patterns in a single evidence map. Therefore, this scoping review aims to systematically map the multidimensional evidence landscape of GI POCUS in the management of enteral nutrition in critically ill patients, addressing current knowledge gaps and informing standardized clinical translation.

## 2 Methods

### 2.1 Design

A protocol was developed *a priori* in accordance with the JBI scoping review methodology (15); however, it was not prospectively registered or published, and no publicly verifiable dated document is available. Scoping reviews serve as valuable tools for comprehensively exploring concepts and related dimensions through a rigorous and transparent methodology, ensuring trustworthy results. The process comprises five sequential stages: research question formulation, identification of relevant literature, systematic study selection, data extraction and charting, and culminating in the collation, synthesis, and reporting of findings. This study is reported in accord with the PRISMA-ScR (16) reporting guideline. This review centers on three core research questions:

1. What specific parameters are evaluated using GI POCUS during enteral nutrition management in critically ill patients?
2. For what distinct clinical objectives and application scenarios are these GI POCUS parameters utilized?
3. What clinical outcomes and predictive dimensions associated with GI POCUS are reported?

### 2.2 Eligibility criteria

Based on the JBI methodology (17), the Population, Concept, and Context (PCC) framework was operationalized to set search boundaries (Table 1). We included adult critically ill patients because their hypercatabolic state makes them uniquely susceptible to ENI, explicitly excluding pediatric/pregnant populations due to distinct physiological guidelines. The Concept covered GI POCUS applications (both morphological and functional/hemodynamic) used specifically to guide feeding or predict ENI. Studies using ultrasound purely for anatomical diagnosis without nutritional relevance were excluded. Finally, the Context was strictly limited to ICUs, as EN management and POCUS implementation are highly protocolized in this setting and heavily confounded by critical care interventions. To align with broader systematic review reporting standards (such as PICOS), the PCC framework elements were systematically mapped to corresponding PICOS dimensions (Table 1).

**Table 1:** Eligibility criteria based on the PCC framework.

PCC element	PICOS equivalent	Inclusion criteria	Exclusion criteria
Population	Population	Adult critically ill patients (age≥18 years).	Pregnant or lactating women.
Concept	Index Concept	Studies evaluating GI POCUS for either: (1) guiding/managing enteral nutrition delivery protocols, or (2) predicting ENI or clinical prognosis.	Studies utilizing ultrasonography solely for anatomical diagnosis without reference to enteral feeding or feeding tolerance.
	Comparator	For comparative interventional studies: routine care, blind syringe aspiration, or standard non-ultrasound protocol controls. For predictive/diagnostic cohorts: non-ENI or surviving reference groups. For purely descriptive mapping studies: a formal comparator was not required.	Interventional studies evaluating clinical efficacy endpoints that lacked an identifiable control or comparative baseline; purely descriptive studies detailing GI POCUS parameters or technical protocols were not excluded based on the absence of a comparator.
	Outcomes	EN delivery efficiency, gastrointestinal complications, nutritional status, ICU length of stay, mortality, and predictive AUCs.	Studies with unextractable outcome data.
Context	Study Design	Intensive Care Units across all subspecialties; Interventional trials (RCTs, NRCTs) and observational cohorts.	Department of Anesthesiology / Operating Rooms; General non-ICU hospital wards; Community / Outpatient clinics; Animal or *in vitro* studies.

EN, enteral nutrition; ENI, enteral nutrition intolerance; GI POCUS, gastrointestinal point-of-care ultrasound; ICU, intensive care unit; NRCT, non-randomized controlled trial; PCC, Population–Concept–Context; PICOS, Population–Index Concept–Comparator–Outcomes–Study design; RCT, randomized controlled trial.

In addition to the PCC framework, we applied the following exclusion criteria: (1) duplicate publications; (2) for interventional studies, the inclusion criterion required a reproducible ultrasound measurement protocol and a stated link between ultrasound findings and feeding decisions; it did not require a fully operationalized feeding-rate titration algorithm; (3) studies with inaccessible full texts or incomplete data that precluded extraction; and (4) publications in languages other than English or Chinese.

### 2.3 Search strategy

We systematically searched six English databases (PubMed, Embase, Cochrane Library, CINAHL, Scopus, Web of Science) and four Chinese databases [China National Knowledge Infrastructure (CNKI), Chinese Science and Technology Periodical Database (VIP), WanFang Data, and Chinese Biomedical Literature Database (Sinomed)] from inception through June 18, 2026 (including ahead-of-print and early-access citations), employing Boolean operators to combine subject headings and free-text terms. Corresponding Chinese terminology was utilized for Chinese databases, with full search strategies detailed in the Supplementary file 1. For each database, the full strategy is reported including platform, coverage period, execution date, field-specific syntax, limits, and number of hits; apparent platform-derived date limits reflect source coverage rather than artificial restrictions.

### 2.4 Literature screening

Our comprehensive search strategy initially identified 5,123 records from 10 electronic databases (Figure 1). The retrieved records were imported into EndNote 20.0 for duplicate removal (*n* = 2,212); an additional 48 records were identified through citation searching of included reviews. Thereafter, 2,911 records underwent title/abstract screening, of which 2,535 were excluded. Of 424 reports sought for retrieval (376 from the database search and 48 from citation searching), 6 were not retrieved. Subsequently, 418 reports underwent full-text eligibility assessment by both researchers, of which 369 were excluded for the following reasons: tube-placement-only studies (*n* = 38), absence of a reproducible ultrasound protocol (*n* = 95), English-Chinese publication duplication (*n* = 21), non-target populations (*n* = 149), and narrative reviews (*n* = 18); the 48 records identified through citation searching were excluded because these duplicate records had already been captured in the database search. This process culminated in 49 unique studies (including 36 interventional studies and 13 observational studies) qualifying for final inclusion (18).

**Figure 1:**
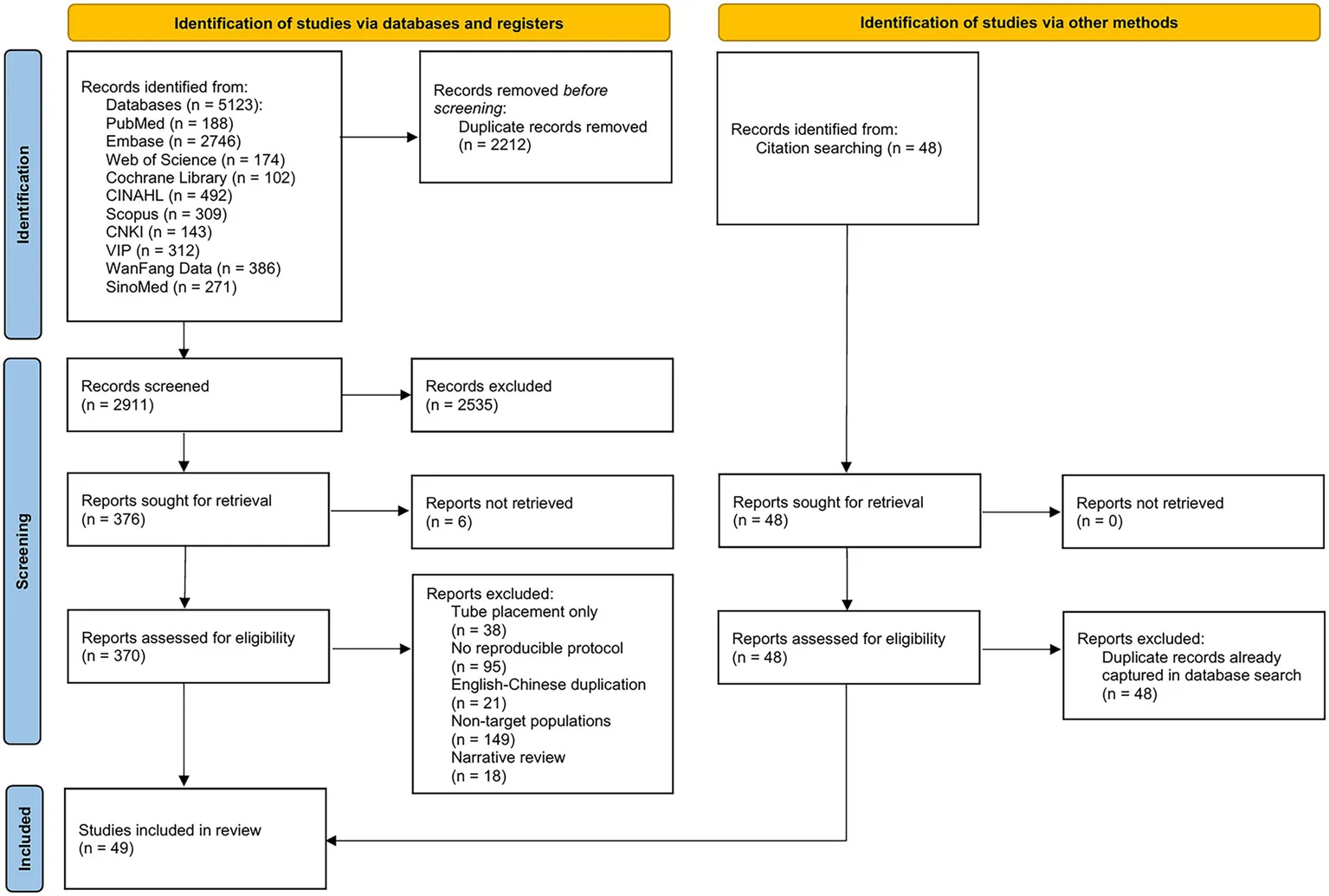
Study selection PRISMA flowchart. CNKI, China National Knowledge Infrastructure; VIP, Chinese Science and Technology Periodical Database; SinoMed, China Biological Medicine Literature Database.

Title/abstract screening and full-text screening were performed independently by two reviewers. A calibration exercise was conducted on a 10% subset before formal screening, and disagreements were resolved by discussion or adjudication by a third reviewer. Chinese-language records were screened by native Chinese-speaking reviewers; data from Chinese studies were extracted in the original language and translated for synthesis, with translation verified by a second bilingual reviewer. Citation chasing and reference-list screening of included reviews did not identify any additional eligible studies beyond those retrieved from the database searches. For six records with unavailable full texts, attempts were made through institutional access, interlibrary loan, and author contact; all attempts were unsuccessful, and these records were excluded with reasons documented.

### 2.5 Data items and charting

Two researchers independently extracted data from each eligible study using a data-charting form that was pilot-tested and calibrated by the review team: two reviewers independently trialed the draft form on 10 studies selected to represent both interventional and predictive designs, compared their entries, clarified variable definitions, revised the affected fields, and then applied the finalized form independently to all included studies; newly added fields were retrospectively completed for previously charted studies. Because no formal psychometric validation was performed, the form is described as calibrated rather than validated. The form collected: authors, publication year, country, study design, sample size, population characteristics, funding source, conflict of interest declarations, ultrasound metrics (including ultrasonographic indicators, evaluation frequency and specific GI POCUS-guided plan), outcome measures, patient position, operator profession, blinding. For predictive studies, we additionally extracted cohort role, AUC, 95% confidence intervals and their provenance, calibration, sensitivity/specificity, and optimal threshold. ENI definitions were recorded verbatim from each primary study. Discrepancies were resolved through consensus discussions, with arbitration by a third researcher when required.

### 2.6 Synthesis of results

To address our three core research questions, we synthesized data across multiple dimensions, detailing GI POCUS parameters, clinical strategies, predictive thresholds, and intervention outcomes for both guiding EN and predicting ENI or prognosis. The following figures were generated to visualize the synthesized evidence, all based on actual data extracted from the included studies and documented in the supplementary extraction tables. The PRISMA flow diagram (Figure 1) was generated using the official PRISMA 2020 flow diagram template (Word version). The anatomical diagram of GI POCUS indicators (Figure 2), the illustration of the GRV-guided four-zone framework for EN management (Figure 3), and the hypothesis-generating conceptual framework for GI POCUS-guided enteral nutrition management (Figure 4) were constructed using Microsoft PowerPoint 2021. The Sankey diagram (Figure 5) and the descriptive AUC evidence map (Figure 6) were generated in R: Figure 5 was constructed using the networkD3 package (v0.4.9) to trace the flow of evidence from geographic origins to clinical objectives and assessment sites, and Figure 6 was produced using the ggplot2 package (v3.4.4). The outcome-domain reporting-frequency map (Figure 7) was generated in Python 3 using the matplotlib library. Data extraction and management were performed in Microsoft Excel 2021.

**Figure 2:**
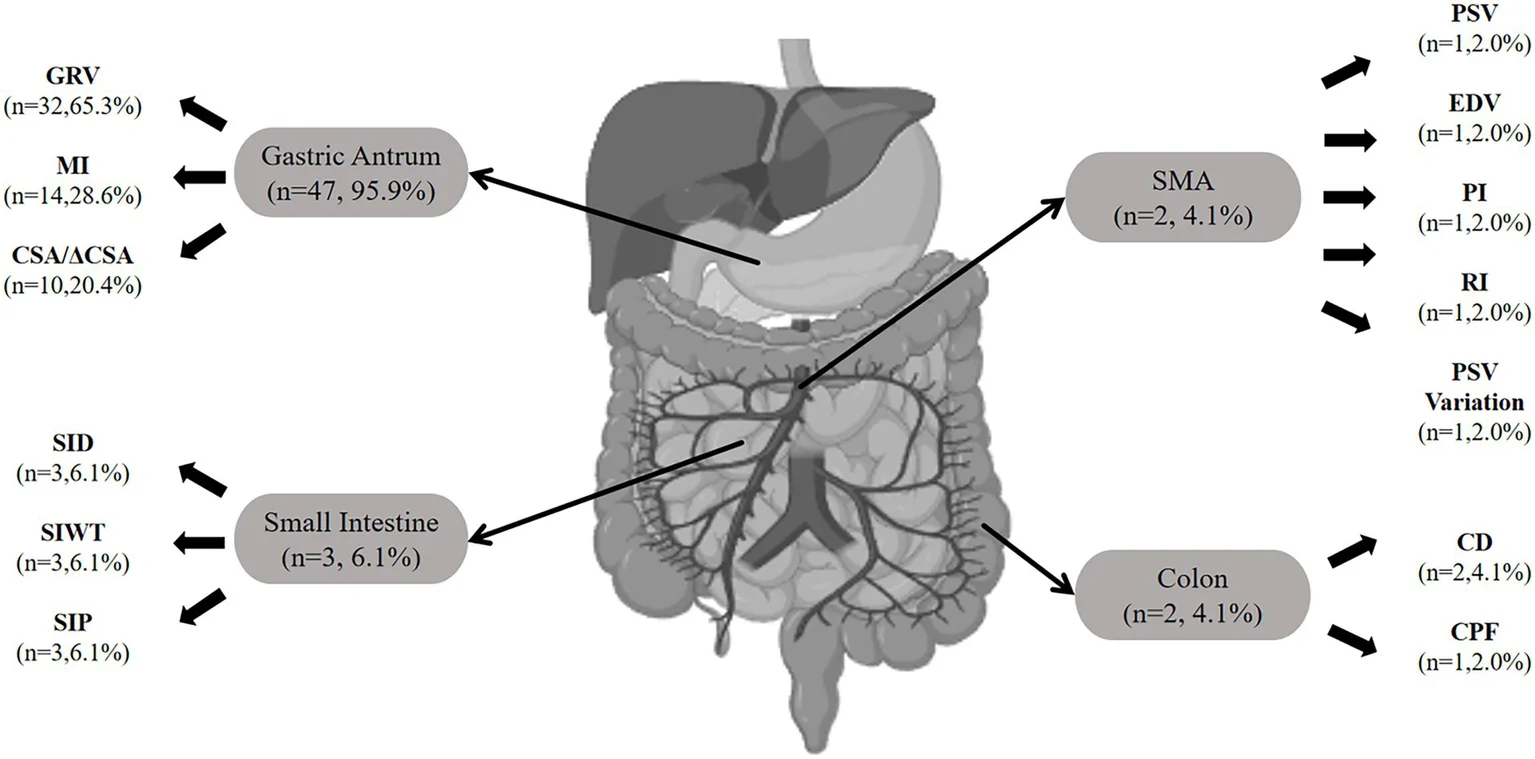
Indicators of GI POCUS used in critical illness. GRV, Gastric Residual Volume; MI, Motility Index; CSA, Cross-Sectional Area; CD, Colon Diameter; SID, Small Intestine Diameter; SIWT, Small Intestine Wall Thickness; SIP, Small Intestine Peristalsis; SMA, Superior Mesenteric Artery; PSV, Peak Systolic Velocity; EDV, End Diastolic Velocity; PI, Pulsatility Index; RI, Resistance Index.

**Figure 3:**
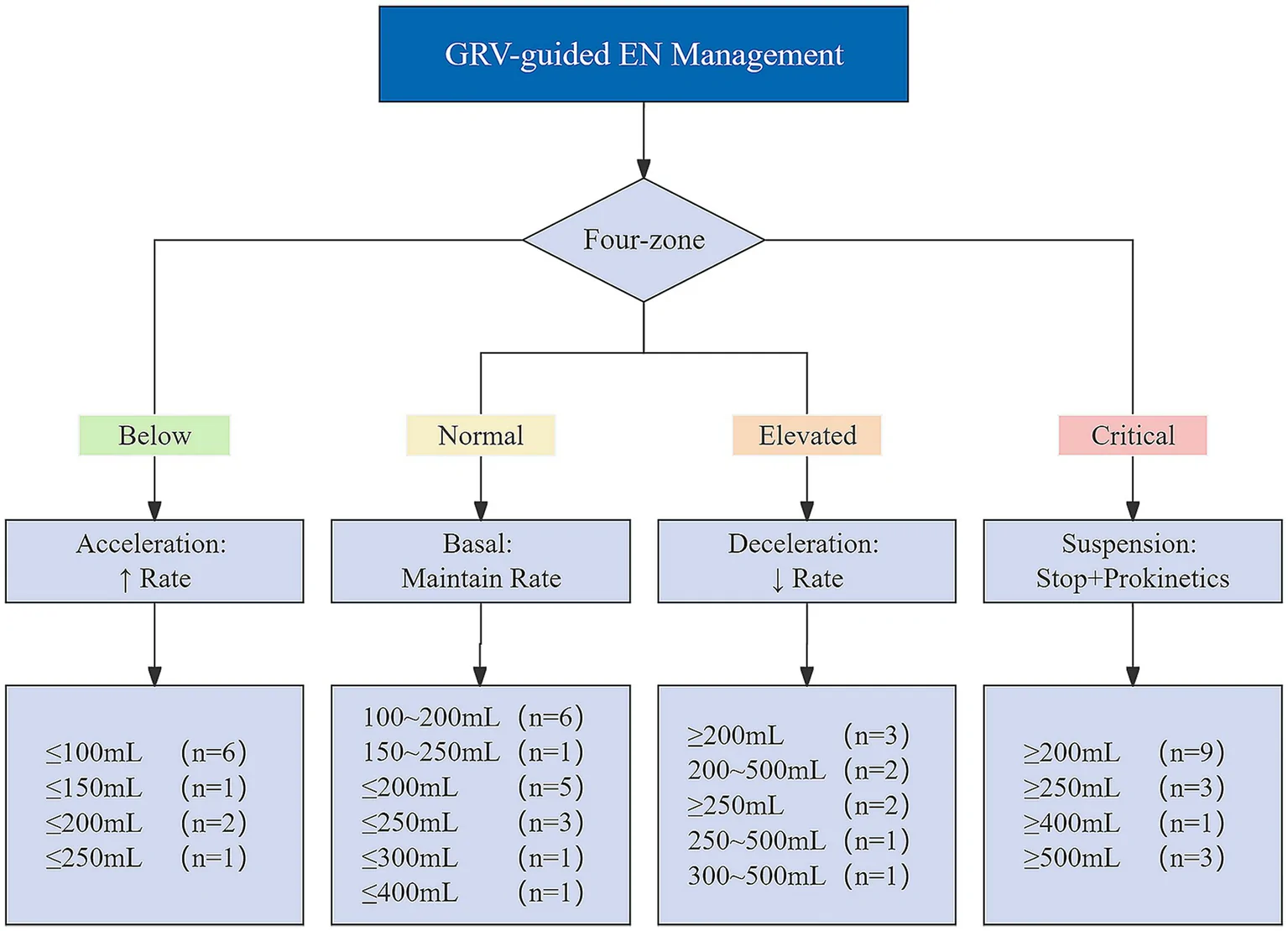
The illustration of GRV-guided four-zone framework for EN management. GRV, Gastric Residual Volume; EN, Enteral Nutrition.

**Figure 4:**
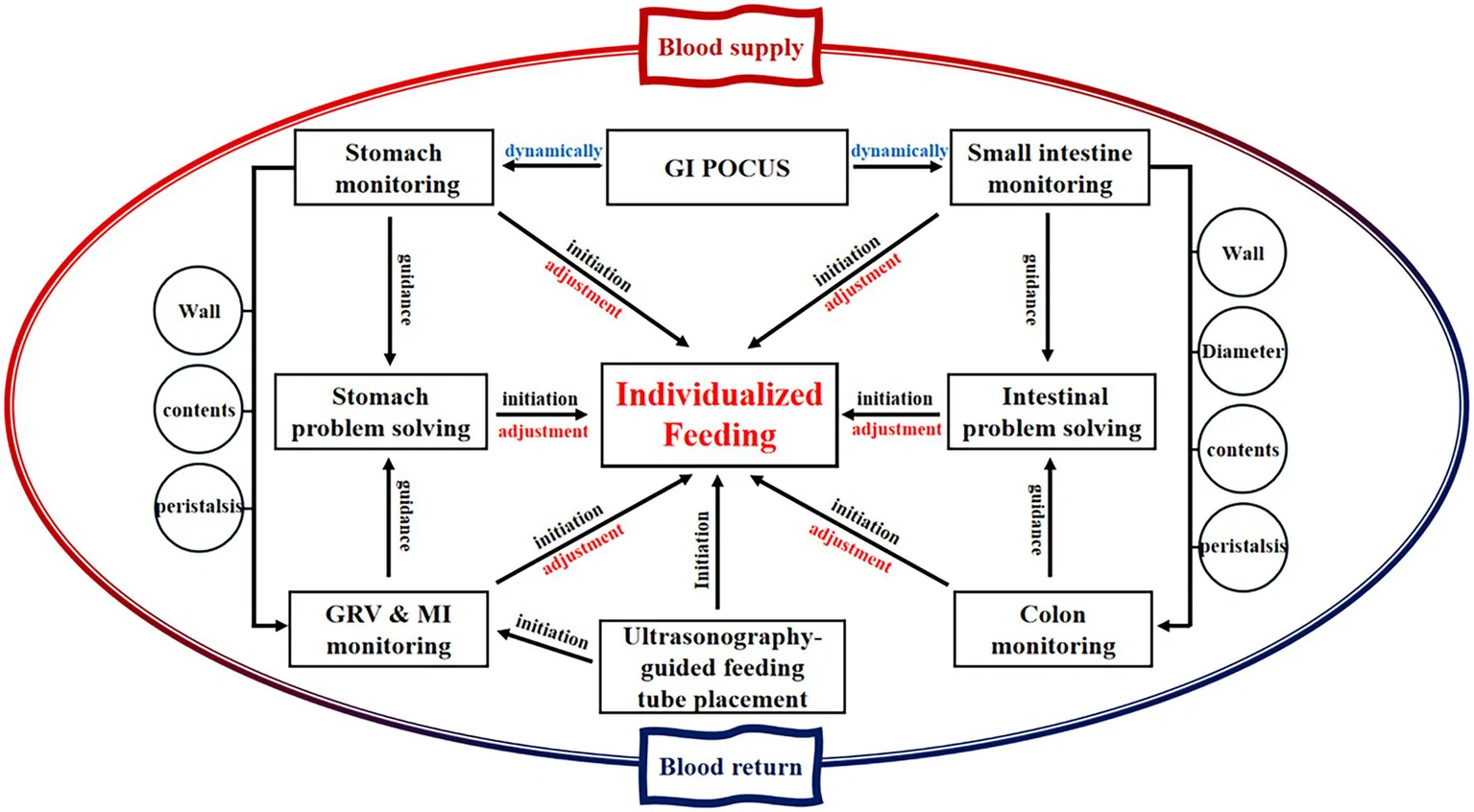
Hypothesis-generating conceptual framework for GI POCUS-guided enteral nutrition management. GRV, Gastric Residual Volume; MI, Motility Index; GI POCUS, gastrointestinal point-of-care ultrasound.

**Figure 5:**
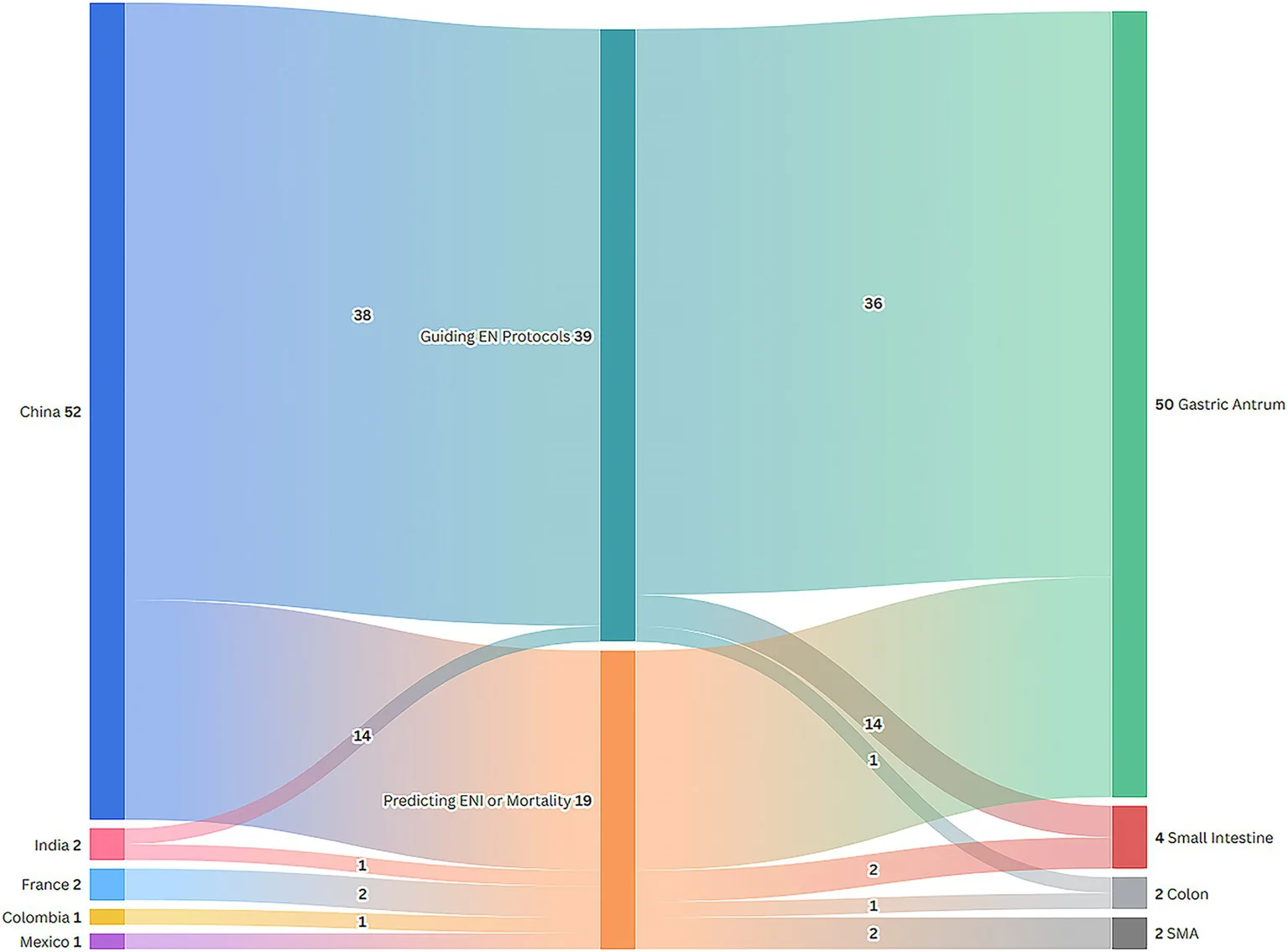
Sankey diagram mapping the evidence flow of GI POCUS research. GI POCUS, Gastrointestinal Point-Of-Care Ultrasound; EN, Enteral Nutrition; ENI: Enteral Nutrition Intolerance; SMA, Superior Mesenteric Artery. Nodes from left to right indicate geographical origins, clinical objectives, and anatomical targets. Band width is proportional to the number of evidence links, not the number of unique studies (*n* = 58 links from 49 unique studies; 39 links for guiding EN protocols and 19 links for predicting ENI/mortality).

**Figure 6:**
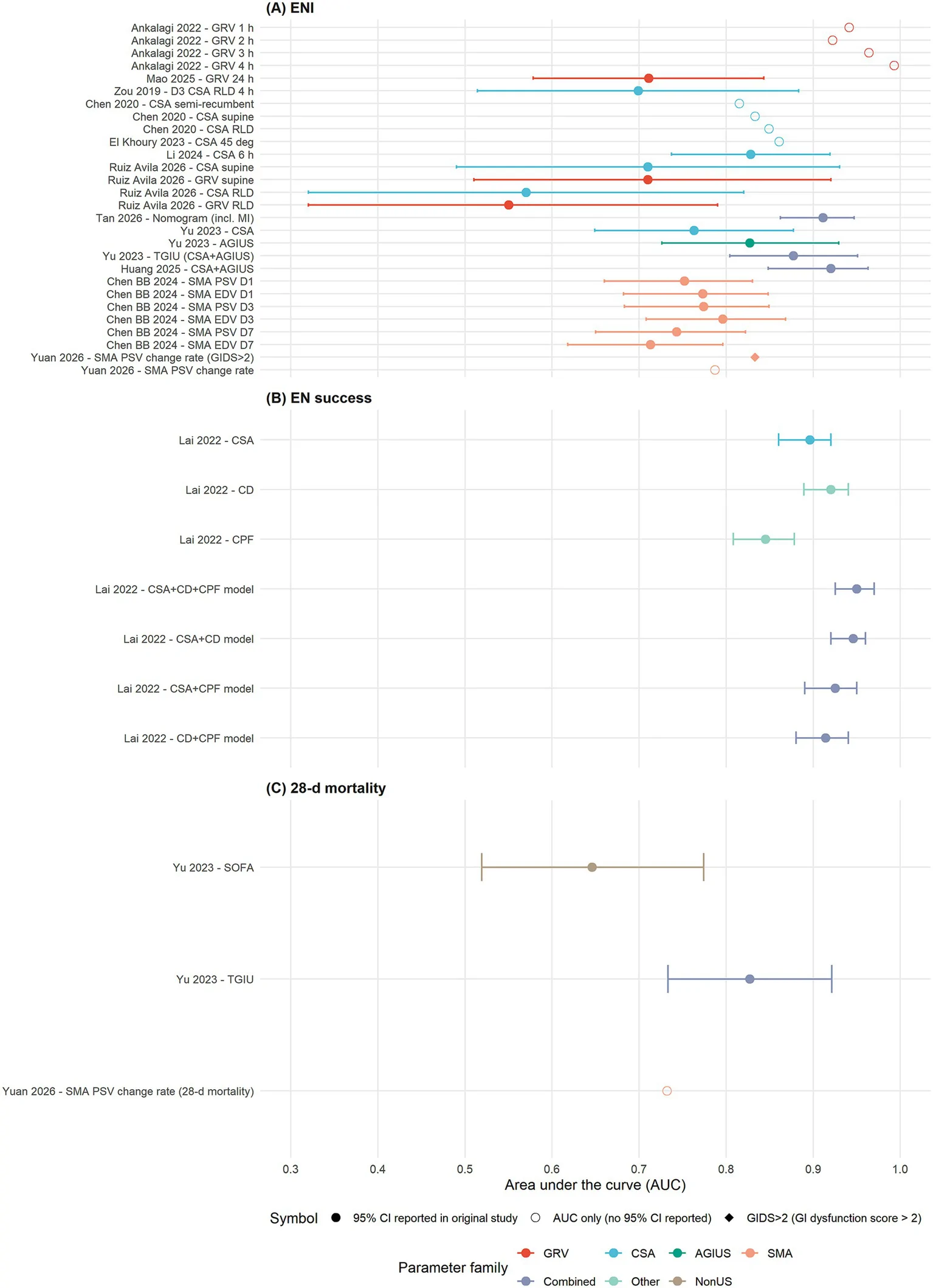
Descriptive map of individual AUC estimates for GI POCUS parameters reported in the included studies. **(A)** ENI (enteral nutrition intolerance) outcomes; **(B)** EN success; **(C)** 28-day mortality. AGIUS, Acute Gastrointestinal Injury Ultrasonography Score; AUC, Area Under the Curve; CD, Colon Diameter; CPF, Colonic Peristaltic Frequency; CSA, (Gastric Antrum) Cross-Sectional Area; D1: Day 1; D3: Day 3; D7: Day 7; EDV, End-Diastolic Velocity; EN, Enteral Nutrition; ENI, Enteral Nutrition Intolerance; GI POCUS, Gastrointestinal Point-of-Care Ultrasound; GIDS, Gastrointestinal Dysfunction Score; GRV, Gastric Residual Volume; MI, Motility Index; Nomogram: Nomogram-based combined prediction model (including the motility index); NonUS, Non-ultrasound parameter; PSV, Peak Systolic Velocity; RLD, Right Lateral Decubitus; SMA, Superior Mesenteric Artery; SOFA, Sequential Organ Failure Assessment; TGIU, Transabdominal Gastro-Intestinal Ultrasonography. Each symbol represents one parameter as evaluated within one included study, not one study as a whole; estimates from the same study are statistically correlated and should not be interpreted as independent evidence units. Symbols are color-coded by parameter family (GRV, CSA, AGIUS, SMA, combined models, and other sonographic parameters); closed and open circles indicate whether a 95% confidence interval was reported in the original study, and the diamond highlights the estimate for GIDS>2. Estimates are displayed descriptively without ordering or pooling; no pooled effect estimate was calculated, and the plot does not establish the predictive value of any parameter.

**Figure 7:**
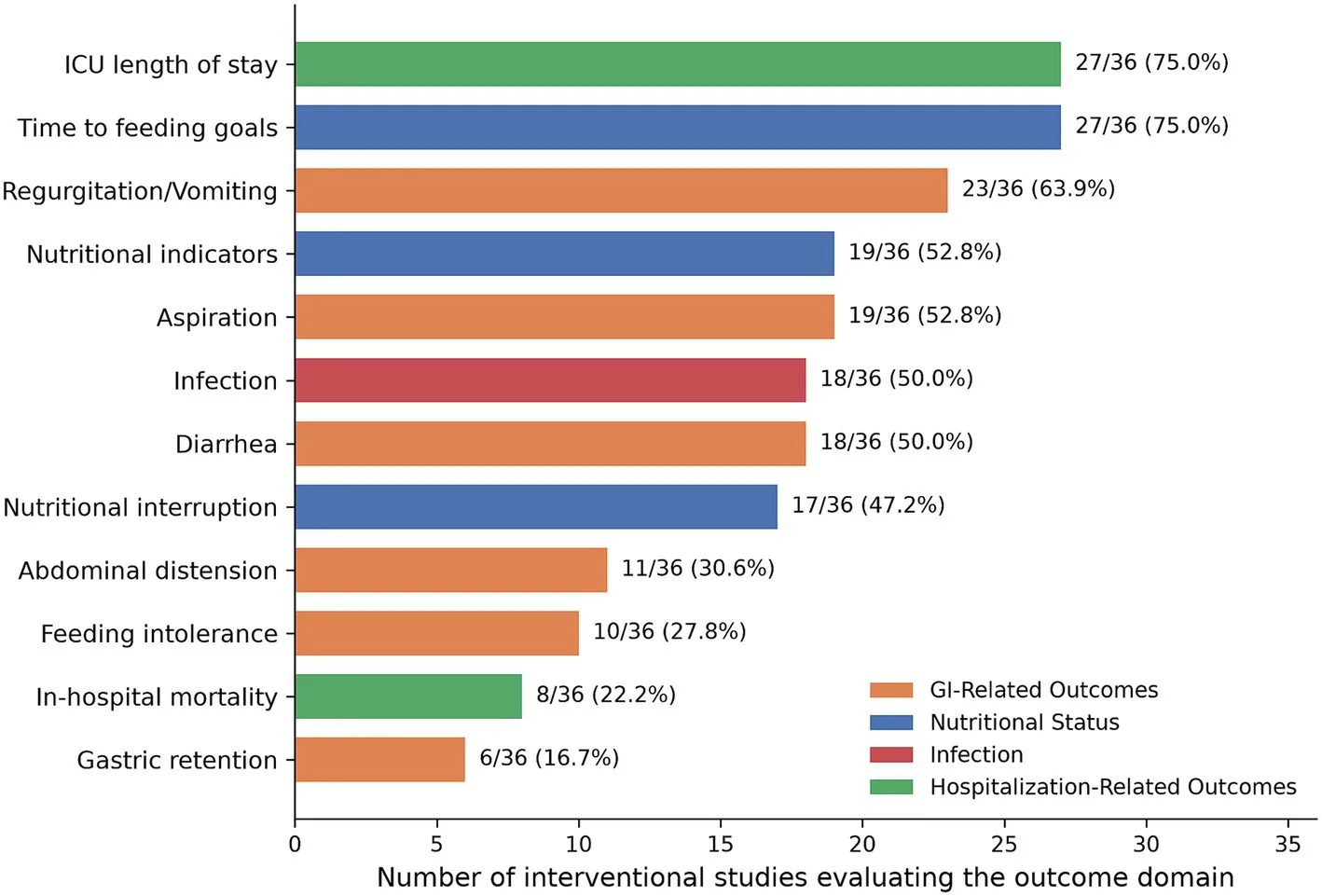
Frequency of outcome-domain evaluation across the 36 interventional studies. GI-Related, Gastrointestinal-Related; ICU, intensive care unit. Bars are colored by outcome category: orange, GI-Related Outcomes (Regurgitation/Vomiting, Aspiration, Diarrhea, Gastric retention, Abdominal distension, Feeding intolerance); blue, Nutritional Status (Nutritional indicators, Nutritional interruption, Time to feeding goals); red, Infection; green, Hospitalization-Related Outcomes (ICU length of stay, In-hospital mortality).

## 3 Results

### 3.1 Characteristics of included studies

A total of 49 unique studies were included in this scoping review. To map the multi-faceted applications of GI POCUS, studies were categorized based on their primary design objectives: 36 studies (19–54) evaluated interventional feeding protocols, and 13 studies (55–67) focused on diagnostic or predictive cohorts (summarized in Supplementary file 2). Of note, 3 interventional studies (43, 52, 53) incorporated a secondary predictive cohort analysis to evaluate the performance of ultrasound parameters for enteral feeding intolerance; consequently, these 3 dual-design studies are represented in both interventional and observational summary tables (totaling 36 interventional and 16 observational studies across tables). Throughout the Supplementary file 2, interventional studies are coded INT-01 through INT-36 and observational/predictive studies OBS-01 through OBS-16; dual-purpose studies are additionally identified by cell shading.

Publication years show a marked temporal trend: All but one study (published in 2011) appeared from 2016 onward (*n* = 48, 98.0%), with a marked concentration in recent years: 43 studies (87.8%) were published between 2019 and 2026, reaching a peak in 2022 (*n* = 10, 20.4%). Geographically, the 49 studies were conducted in China (*n* = 43, 87.8%), India (*n* = 2), France (*n* = 2), Colombia (*n* = 1), and Mexico (*n* = 1). To map the design characteristics of the available literature, studies were categorized by design as 27 randomized controlled trials, 9 non-randomized controlled trials, and 13 independent observational studies.

Across the 49 included studies, the critically ill populations included diverse clinical subgroups. The most frequently studied groups were mechanically ventilated patients (*n* = 9), followed by patients with sepsis (*n* = 6), neurocritical conditions (*n* = 5), prone-positioned ventilated patients (*n* = 2) and geriatric ICU patients (*n* = 2). Specific populations represented by a single study included: acute gastrointestinal injury patients, post-lung-transplant, rheumatic heart disease with cachexia, acute exacerbation of chronic obstructive pulmonary disease ventilated patients, neurocritical ventilated patients, moderately severe acute pancreatitis, severe traumatic brain injury, severe pneumonia, acute traumatic complete cervical spinal cord injury, and spontaneous intracerebral hemorrhage patients. The remaining 15 studies included general critically ill cohorts without a specified predominant diagnosis.

Sample size distributions were as follows: among the 36 interventional studies, experimental group sample sizes ranged from 18 to 110 (median 38.5, IQR 30–52), and control group sample sizes ranged from 19 to 106 (median 38.5, IQR 30–52). Among the 16 observational study records (including 3 dual-design studies), sample sizes ranged from 30 to 513 (median 76, IQR 50–150); the 13 independent observational studies had a median sample size of 92 (range 42–513).

With respect to acquisition variables, patient position during scanning was reported in 36 of 49 included studies (24 of 36 interventional and 12 of 13 observational/predictive studies), predominantly using right lateral decubitus, supine, or head-of-bed-elevated positions. The feeding state at scanning was documented in all 16 studies contributing predictive analyses (13 observational cohorts plus 3 dual-purpose interventional studies), ranging from fasting baseline to predefined post-feeding time points. Operator profession and prior training were reported in only 18 of 49 studies (36.7%); among these, three interventional studies explicitly designated nurses as examiners (19, 24, 49), with one additionally requiring physician adjudication when scans disagreed (49), whereas the other 15 studies used critical care physicians, radiologists, certified POCUS experts, or unlabeled investigators; no observational/predictive study used nurses. Blinding was infrequently described (3 of 49 studies).

Funding sources and conflict of interest (COI) declarations were extracted for all 49 included studies (Supplementary file 2). Of these, 29 (59.2%) reported funding sources, predominantly national and provincial natural science foundations and health commission research projects; 20 studies (40.8%) explicitly declared no COI, while 29 studies (59.2%) did not report COI information, the latter being characteristic of Chinese-language journals.

### 3.2 Indicators of GI POCUS

Across the 49 included studies, the evaluated GI POCUS parameters can be systematically categorized based on their targeted anatomical locations: the gastric antrum, small intestine, colon, and superior mesenteric artery (SMA). The gastric antrum was the most frequently assessed region, with parameters including GRV, motility index (MI), and cross-sectional area (CSA). Specifically, 22 studies assessed GRV exclusively (19–36, 53, 54, 57, 61), 7 focused solely on MI (37–42, 65), 5 evaluated only CSA (43, 55, 56, 60, 64), 6 concurrently assessed MI and GRV (44–48, 51), and 1 assessed GRV and CSA (66).

Assessments extending to the lower gastrointestinal tract incorporated the colon, evaluating colon diameter (CD) and colonic peristaltic frequency (CPF), and the small intestine, focusing on small intestine diameter (SID), wall thickness (SIWT), and peristalsis (SIP). 3 studies (50, 52, 62) implemented the Acute Gastrointestinal Injury Ultrasound Score (AGIUS) utilizing these small intestine metrics, while 2 studies (49, 58) employed a multi-regional approach by combining gastric parameters (GRV/CSA & MI) with colonic parameters (CD & CPF).

Furthermore, vascular assessment via SMA hemodynamics emerged as a specialized focus; 2 studies (63, 67) exclusively evaluated SMA perfusion, calculating the Pulsatility Index (PI), Resistance Index (RI) and PSV Variation based on Peak Systolic Velocity (PSV), End-Diastolic Velocity (EDV), and Time-Averaged Mean Velocity (TAMV; PI = [PSV - EDV] / TAMV; RI = [PSV - EDV] / PSV; PSV Variation = (PSV_day3_ - PSV_day1_) / mean PSV_day1-day3_), to investigate the predictive roles of these hemodynamic parameters in ENI. The specific distribution of these parameters across their respective anatomical regions is visually summarized in Figure 2.

### 3.3 Clinical objectives and application scenarios

The application scenarios of GI POCUS in current research diverge into two primary clinical objectives: guiding EN protocols, predicting ENI and clinical prognosis. Furthermore, to comprehensively map the complex evidence flow connecting geographical origins, these anatomical foci, and their subsequent clinical objectives, a Sankey diagram was constructed (Figure 5).

#### 3.3.1 Guiding enteral nutrition protocols

Among the 36 interventional studies implementing GI POCUS guided EN protocols, feeding rate adjustment strategies were governed by either single-parameter monitoring (GRV or MI) or multi-parameter integration (combining gastric and intestinal ultrasonography).

For protocols relying exclusively on GRV (*n* = 20; 16 RCTs and 4 NRCTs), a recurrent “four-zone” pattern was identified among studies reporting explicit protocols (Figure 3): (1) Acceleration zone, where sub-threshold GRV triggered rate increases; (2) Basal rate zone, maintaining infusion speeds for within-range GRV; (3) a deceleration zone, prompting rate reductions for elevated GRV; and (4) a suspension zone, requiring feeding cessation and prokinetic administration for critical thresholds. One of these studies (53), alongside a study that evaluated CSA alone (43), did not detail a specific enteral nutrition adjustment protocol. These two studies were retained because they reported a reproducible ultrasound measurement protocol and explicitly linked the findings to feeding management or ENI prediction; they simply did not report the detailed rate-adjustment ladder that characterizes the four-zone protocols described above.

Critical transition values demarcating basal rate from deceleration/suspension zones predominantly clustered at 200 mL or 250 mL. For suprathreshold GRV, two distinct management strategies emerged: immediate feeding suspension with scheduled re-evaluation, or implementation of transitional deceleration zones prior to suspension. Two studies (27, 33) enhanced protocol granularity through dual-tiered deceleration zones. Conversely, subthreshold GRV activated acceleration protocols with continuous rate escalation until maximum target delivery rate was achieved.

In contrast, protocols guided solely by MI demonstrated a more consistently reported stratification: MI served as a direct velocity regulator, dictating infusion rates at 20–30 mL/h (MI < 0.4), 40–60 mL/h (0.4 ≤ MI < 0.8), and >70 mL/h (MI ≥ 0.8). Furthermore, two of these studies incorporated supplementary clinical scoring tools to cross-validate ultrasonographic findings prior to escalating delivery rates; one utilized a 3-symptom Feeding Intolerance Scale (41), while the other adopted a comprehensive 7-item Enteral Nutrition Tolerance Score (42).

Multi-parameter integrated protocols generally combine ultrasonographic evaluations of the stomach with those of the intestines, thereby seeking to address the limitations associated with isolated single-parameter monitoring. These studies are broadly classified into two categories: combining two gastric parameters (GRV and MI) or pairing GRV with intestinal indicators. Among studies evaluating the combination of GRV and MI (*n* = 6; 5 RCTs and 1 NRCT), protocols consistently utilized GRV as a primary safety gatekeeper to rule out gastric retention (confirming that GRV remained within the “Acceleration zone”), after which the MI was directly applied to determine the feeding infusion rate. One randomized controlled trial further extended this multi-parameter approach by incorporating CD and SID assessments to evaluate bowel distension (49). Similarly, two studies guided feeding rate adjustments through combined GRV and AGIUS monitoring (50, 52), using AGIUS as a composite metric, incorporating small intestine diameter, wall thickness, and peristalsis, to modulate feeding progression.

In these comparative trials, the majority of control groups utilized traditional syringe aspiration to quantify gastric residual volume. Because syringe aspiration is restricted to evaluating GRV alone, the protocols in the control groups were exclusively anchored to GRV thresholds, mirroring the GRV-dependent components observed in the experimental groups. Complete and detailed protocols are documented in Supplementary file 2.

Ultrasound monitoring intervals exhibited substantial heterogeneity. More than half of the interventional trials (*n* = 20) adopted intensive monitoring schedules ranging from every 4 to 6 h. Less frequent assessments included once daily (*n* = 7) or twice daily (*n* = 4) protocols. The remaining studies employed 8 h intervals (*n* = 1), specific intermittent days (n = 1), or did not specify the frequency (*n* = 3).

#### 3.3.2 Predicting ENI and clinical prognosis

Beyond active feeding guidance, 13 observational studies (55–67) and 3 interventional studies (43, 52, 53) utilized GI POCUS to establish preemptive sonographic thresholds prior to overt clinical symptoms.

For isolated gastric metrics, observational studies identified a static GRV ≥ 216.7 mL (measured in the right lateral position at 4 h post-EN initiation) as its study-derived cut-off for predicting ENI (57). Contrastingly, two recent studies reported substantially lower and varied threshold values: one identified a GRV cutoff of 9.40 mL (53), whereas another observed a cutoff of 33.3 mL (66), these unusually low values arose from different time points, and body positions rather than transcription errors; Furthermore, another study demonstrated that an absolute GRV ≥ 250 mL failed to independently predict mortality or prolonged ICU stay, merely indicating a delay in target feeding achievement (61). Predictive thresholds for static gastric antral CSA clustered within a relatively narrow range across several studies, clustered around 7.092 cm^2^ (day 3, right lateral) (55), 7.20 cm^2^ (12 h, semi-upright) (60), 7.835 cm^2^ (4 h, supine) (43). However, notable deviations emerged in two studies reporting markedly lower threshold values. Specifically, one study documented position-dependent CSA cutoffs of 3.917 cm^2^ (semi-upright), 3.395 cm^2^ (supine), 4.402 cm^2^ (right lateral) (56). Similarly, another study identified a lower CSA threshold of 5.48 cm^2^ (66).

Expanding beyond static morphological evaluations, two studies highlighted the predictive value of dynamic gastric functional assessments for ENI. Specifically, a fractional change in CSA (ΔCSA) cut-off of 52% at 60 min was a highly specific predictor for ENI (59), whereas a higher MI on Ventilation Day 3 was identified as an independent protective factor against ENI (65).

Several studies integrated dual assessments of the stomach and intestine to evaluate their combined predictive efficacy for ENI. One study demonstrated that a combination of CSA ≤ 9 cm^2^, CD ≤ 2.8–2.9 cm, and CPF > 3 bpm were associated with favorable enteral feeding tolerance in that cohort (58). Furthermore, another study (62) established that a CSA > 6 cm^2^ combined with an AGIUS score > 3.5 were associated with first-week ENI in that cohort, whereas higher critical values (CSA > 10.2 cm^2^, AGIUS > 4.5) were associated with a significantly elevated 28-day mortality risk in that cohort. In contrast, a recent study observed divergent thresholds for predicting ENI, reporting a combined cut-off of a CSA of 8.90 cm^2^ and an AGIUS of 1.

Finally, the predictive capacity of SMA hemodynamics (including PSV, EDV, PI, and RI) as well as their longitudinal variations for ENI was explored and showed preliminary associations, although specific numerical cutoffs were not explicitly reported in those studies (63, 67).

Individual study AUC estimates (with 95% confidence intervals where reported) are displayed descriptively in three panels according to the predicted outcome: (A) enteral nutrition intolerance (ENI), including one analysis predicting a gastrointestinal dysfunction score > 2 (GIDS > 2) that is highlighted separately; (B) enteral feeding success; and (C) 28-day mortality (Figure 6). All 38 extracted AUC estimates were derived from single-cohort derivation studies; none of the 13 contributing studies reported an independent external validation cohort; multiple estimates from the same study (reflecting different parameters, positions, time points, or models) are statistically correlated and should not be interpreted as independent evidence units. Among the reported estimates, point values frequently ranged around or above 0.70, with substantial heterogeneity across predictors, patient positions, ENI definitions, and thresholds. Confidence intervals were extracted directly from primary publications where reported; where intervals were not available in the source, they are not plotted. No pooled estimate was calculated. Consistent with scoping review methodology, these findings are presented descriptively and do not constitute validated predictive performance.

### 3.4 Outcome domains and endpoint reporting patterns

Outcomes from the 36 interventional studies were categorized into four domains: (1) gastrointestinal-related outcomes (regurgitation/vomiting, aspiration, diarrhea, gastric retention, abdominal distension, enteral nutrition intolerance); (2) nutritional status (nutritional indicators, nutritional interruption rate, time to achieve feeding goals); (3) infection rates; and (4) hospitalization-related outcomes (ICU length of stay, in-hospital mortality).

Figure 7 presents the frequency with which each outcome domain was evaluated across the 36 interventional studies, irrespective of the direction, magnitude, or statistical significance of between-group differences. Time to achieve feeding goals and intensive care unit length of stay were the most frequently evaluated outcomes (each 27/36, 75.0%), followed by regurgitation/vomiting (23/36, 63.9%), aspiration and nutritional indicators (each 19/36, 52.8%), diarrhea and infection (each 18/36, 50.0%), nutritional interruption (17/36, 47.2%), abdominal distension (11/36, 30.6%), feeding intolerance (10/36, 27.8%), in-hospital mortality (8/36, 22.2%), and gastric retention (6/36, 16.7%). Effect estimates with measures of precision were not uniformly reported across primary studies and are therefore not displayed. No pooling of effect magnitudes, directions, or confidence intervals was performed, due to (1) substantial heterogeneity in the operational definitions of gastrointestinal outcomes across studies, (2) variability in study designs, intervention protocols, and patient populations, and (3) the scoping review methodology, which prioritizes evidence mapping over quantitative synthesis.

Considerable heterogeneity was observed in the operational definitions used across studies for specific gastrointestinal symptoms. Detailed definitions extracted from the literature were compiled and are presented in Supplementary file 3.

## 4 Discussion

Current EN management in critically ill patients primarily relies on monitoring gastrointestinal symptoms such as vomiting, abdominal distension, and diarrhea (68). However, as highlighted by the substantial heterogeneity in outcome definitions identified in this review, this symptom-based approach is limited not only by subjectivity but also by a lack of standardization, leading to delayed or inconsistent interventions. This scoping review shows how GI POCUS may complement symptom-based assessment by providing objective, real-time imaging information of gastrointestinal indicators. By providing quantifiable physiological insights, POCUS may support a shift from reactive symptom management toward more objective, bedside-informed assessment of enteral feeding strategies.

### 4.1 Summary of evidence and study characteristics

Our scoping review mapped 49 studies investigating ultrasound-guided enteral nutrition management in critically ill patients. Geographically, the predominant concentration of included studies originating from China reflects substantial regional research interest in bedside ultrasound protocols. The drivers of this geographic concentration were not formally evaluated in the included literature and were not inferred by the authors; factors such as professional scope of practice, bedside-ultrasound training and certification, equipment availability, nursing staffing, enteral-nutrition protocols, case mix, and ICU resources may differ across health systems and may influence both the conduct of such research and the transportability of its protocols. Accordingly, the mapped protocols cannot be assumed to be directly applicable to other ICUs without local validation. However, because the underlying drivers of this regional focus were not formally evaluated in the included literature, large-scale international multicenter studies across diverse critical care settings remain necessary to establish the broader applicability and generalizability of these GI POCUS protocols.

The included studies demonstrated substantial clinical heterogeneity regarding patient populations. While mechanically ventilated and neurocritical patients were the most common subgroups, the scope extended to specific niches such as prone positioning, lung transplantation, and geriatric care. This diversity illustrates the breadth of populations in which GI POCUS has been explored, but it also highlights the complexity of establishing a universal protocol. Given the substantial heterogeneity in patient populations, ultrasound protocols, and clinical endpoints, we adopted a scoping review methodology to map study designs, interventional strategies, and reporting patterns without conducting formal risk-of-bias assessments or meta-analyses of efficacy.

Our scoping review expands upon recently published evidence syntheses in several meaningful dimensions. Whereas a recent meta-analysis (12) provided pooled diagnostic accuracy estimates strictly for feeding intolerance across diagnostic cohorts, it did not synthesize interventional feeding protocols. Similarly, while recent reviews (13, 14) have explored bedside ultrasound applications in critical care nutrition, our review updates the evidence landscape through June 18, 2026, synthesizing 49 unique studies. More importantly, our work bridges predictive assessment with interventional practice by simultaneously mapping operational feeding adjustment strategies and the outcome-reporting patterns. This positions our review as a comprehensive, protocol-and-outcome evidence map rather than a purely diagnostic summary.

Supplementary file 4 provides a systematic comparison of this review with three recently published evidence syntheses, detailing differences in search end date, databases and languages, population and context, number and types of included studies, anatomical domains, whether interventional protocols were mapped, whether predictive accuracy was assessed, whether risk of bias was evaluated, and the specific incremental contribution of the present review.

### 4.2 Sources and implications of heterogeneity

The marked heterogeneity observed across the included studies can be organized into six domains, each with specific implications for interpretation and synthesis.

(1) Clinical heterogeneity: patient case mix (e.g., mechanical ventilation, prone positioning, sepsis, neurocritical conditions), disease severity, vasoactive and sedative drug use, pre-existing gastrointestinal disease, and the phase of enteral feeding differed markedly; thresholds and effects derived in one subgroup may not transfer to another. (2) Index-test heterogeneity: parameters targeted the gastric antrum, small intestine, colon, or superior mesenteric artery and included static (GRV, CSA), dynamic (MI, ΔCSA), composite (AGIUS), and hemodynamic (PSV, EDV, PI, RI) metrics, which cannot be treated as equivalent measurements. (3) Measurement heterogeneity: body position, and time from feeding to measurement varied across studies, so numerical cut-offs are not interchangeable. (4) Operator heterogeneity: operator profession, prior experience, training, blinding, and inter−/intra-observer reliability were frequently unreported, limiting assessment of reproducibility. (5) Intervention heterogeneity: feeding-rate thresholds, adjustment magnitude, monitoring frequency, prokinetic use, comparator protocols, and co-interventions differed, so the mapped “four-zone” and related patterns are descriptive generalizations of existing protocols rather than standardized algorithms. (6) Outcome and design heterogeneity: ENI definitions, follow-up duration, surrogate versus patient-important outcomes, and study designs (RCT, NRCT, derivation versus validation cohorts) differed.

These six domains explain why thresholds and effects cannot be pooled, why the synthesis was restricted to descriptive mapping, and why external validation and reporting standards are prerequisites for clinical translation (69).

### 4.3 Physiological evolution: from gastric morphology to multi-segmental and hemodynamic assessment

The predominance of studies focusing on GRV reflects its established historical role as the primary indicator of EN in clinical practice (70). In these studies, ultrasonographic measurement typically employed single-plane scanning of the gastric antral cross-sectional area, with GRV calculated using age-specific formulas (71) to dynamically adjust feeding protocols. This practice has been reported to mitigate complications such as regurgitation and aspiration (72). However, while ultrasound-guided GRV assessment may reduce the limitations associated with blind aspiration, relying solely on a “static” volumetric parameter has inherent physiological limitations (73). Physiologically, gastric emptying is a function of antral contractility, driven by the coordinated activity of the proximal gastric pressure pump and distal antral peristaltic pump (74, 75). Consequently, significant gastric distension is a late indicator of dysfunction, signifying that these primary propulsive mechanisms have already failed. Promisingly, nearly a third of the included studies incorporated the MI. This emerging shift from purely morphological assessment to functional assessment is crucial, as capturing real-time antral contraction frequency and amplitude may offer earlier detection of ENI than volume retention alone.

However, a significant “gastric-centric” bias remains evident. Only 5 studies extended their assessment to intestinal parameters (small intestine or colon), and comprehensive scoring systems like AGIUS were rare. Gastrointestinal dysfunction in critical illness is rarely isolated to the stomach; it often involves the entire alimentary tract, comprising acute gastrointestinal injury syndromes (6). Focusing solely on the stomach ignores the physiological reality of distal feedback mechanisms, such as the ‘ileal brake’. Signals from the distal intestine trigger an enterogastric feedback loop that actively inhibits gastric emptying (76). Therefore, elevated GRV may represent a manifestation of downstream intestinal intolerance rather than a primary gastric dysfunction. Future protocols should explore a multi-segmental assessment strategy covering the entire gastrointestinal tract, thereby providing a holistic view of GI patency.

An emerging paradigm within the synthesized evidence is the incorporation of hemodynamic assessment. Physiologically, the functional integrity of the gastrointestinal tract is fundamentally predicated on the maintenance of adequate perfusion pressure and a patent vascular supply (77). While representing a minority of the included studies, emerging hemodynamic assessments have highlighted the PSV of the SMA, particularly its longitudinal dynamic variation, as a promising or potential predictor for gastrointestinal dysfunction (63, 67). Integrating such vascular hemodynamic metrics into bedside POCUS protocols could broaden gastrointestinal assessment toward earlier perfusion-related evaluation.

### 4.4 Outcome-reporting patterns

Time to achieve feeding goals was the most frequently evaluated nutritional metric. Established guidelines (73) emphasize early initiation of enteral nutrition and timely progression toward caloric targets to mitigate energy deficits. Individual studies that reported shorter times to achieve nutritional targets under ultrasound-guided protocols may reflect more individualized feeding titration rather than arbitrary hold-orders, but this pattern cannot be synthesized quantitatively here. It should also be contextualized within evolving clinical paradigms. Recent studies (78, 79) and updated international consensus guidelines (80, 81) caution against rapid full-calorie delivery during the hyperacute phase of critical illness, favoring controlled hypocaloric feeding (6 to 8 kcal/kg/d) to avoid metabolic stress. Within this updated framework, time to achieve feeding goals should be regarded as a process/surrogate outcome: faster target attainment does not by itself establish improvement in patient-important outcomes such as mortality, infection, aspiration, mechanical-ventilation duration, ICU length of stay, or adverse events, and whether it should continue to serve as a primary indicator for evaluating enteral nutrition efficacy remains questionable.

Gastrointestinal-related outcomes were evaluated with variable frequency: regurgitation/vomiting (23/36), aspiration (19/36), diarrhea (18/36), abdominal distension (11/36), feeding intolerance (10/36), and gastric retention (6/36). Their divergent operational definitions are compiled in Supplementary file 3. Beyond definitional heterogeneity, these symptoms have a complex, multifactorial etiology in critical illness. Symptoms such as diarrhea and abdominal distension are heavily confounded by broad-spectrum antibiotic administration, gut dysbiosis, and systemic inflammation, which are pathophysiological mechanisms that sonographic feeding adjustments alone cannot modify.

Hospitalization-related outcomes were evaluated asymmetrically: intensive care unit length of stay was reported in 27 of 36 studies, whereas in-hospital mortality was evaluated in only 8 of 36, indicating that hard endpoints remain under-assessed in this evidence base. One small single-center study reported lower mortality in the ultrasound-guided group (3/31 [9.7%] versus 10/33 [30.3%]; *p* = 0.040). Given the small sample and the sensitivity of this comparison to the analytical method, this isolated finding should not be interpreted as evidence of a mortality benefit. This asymmetry between frequently measured process or surrogate endpoints and sparsely measured hard outcomes is methodologically aligned with the role of GI POCUS as an observational monitoring tool rather than an independent therapeutic agent. Because this review maps outcome-reporting frequency rather than assessing risk of bias or pooling clinical effect sizes, these findings illustrate existing research tendencies rather than proven therapeutic superiority.

### 4.5 Implementation considerations for nurse-performed GI POCUS

Enteral feeding delivery and bedside monitoring are routine nursing responsibilities in the ICU. Three included interventional studies explicitly performed GI POCUS under nursing examiner responsibility; this subsection therefore briefly examines the implementation conditions for nurse-performed GI POCUS, drawing on the mapped evidence and related external literature.

Our findings indicate considerable conservatism in current POCUS protocols, with cutoff thresholds clustering around 200 mL to 250 mL. The SCCM/ASPEN 2016 guideline recommends against routine blind gastric residual volume monitoring in the ICU and, if residual volume is used, against interrupting enteral nutrition for volumes below 500 mL in the absence of other signs of intolerance (73); the ESPEN 2019 guideline suggests delaying enteral nutrition when the gastric aspirate volume exceeds 500 mL per 6 h, in combination with abdominal examination and prokinetic therapy (82). These 500 mL criteria refer to aspirated gastric residual volume and have not been validated as ultrasound-derived thresholds; the lower sonographic cut-offs mapped in this review therefore cannot be directly compared with these recommendations without studies of measurement agreement, calibration, and clinical safety. Whether the low sonographic thresholds used in the mapped studies contribute to unnecessary feeding interruptions or underfeeding is an empirical question that should be evaluated prospectively rather than assumed.

The observed heterogeneity in monitoring intervals, ranging from every 4 h to daily, highlights the need to balance patient safety with nursing workload. Future studies should evaluate whether transitioning from intensive monitoring during the acute phase to a less frequent maintenance schedule remains clinically viable once tolerance is established.

Within the included evidence map, operator profession and prior training were reported in only 18 of 49 table entries (36.7%). Among the 18 entries that reported operator identity, only three interventional studies (19, 24, 49) explicitly used nurses as examiners, and no observational predictive study was performed by nurses. The other 31 entries did not state who performed the scans. The included studies therefore do not by themselves support conclusions about nurse-led practice. Contextual evidence outside the included evidence map indicates that nurse-performed POCUS in adult ICUs can support real-time clinical decisions, with therapeutic management modifications in 26 to 67% of assessments without increased complication rates (83), and that GI POCUS is already used in daily Chinese critical care nursing practice, primarily for gastric residual volume assessment (61.3%), feeding tube placement guidance (53.2%), and enteral nutrition tolerance evaluation (33.1%) (84). These external observations were not used to answer this review’s efficacy or prediction questions. Whether nurse-performed GI POCUS can be integrated into enteral feeding protocols remains an implementation research question requiring evaluation of feasibility, competency frameworks, governance, supervision, diagnostic agreement with reference standards, and patient safety across different settings.

Competency for nurse-performed GI POCUS requires evaluation rather than assumption. In one prospective educational cohort, eight novice bedside nurses without prior ultrasound experience performed gastric ultrasound on healthy volunteers; all participants met the study-specific competency definition after a median of 23 supervised scans, and a learning model estimated approximately 30 scans for 95% success (85). Because this estimate derives from a small, task- and population-specific educational context, it should not be treated as a universal credentialing threshold for GI POCUS in critically ill patients or for intestinal or vascular examinations. The absence of standard operating procedures, quality control systems, and formal mentorship frameworks remains a substantive barrier to real-world implementation (84).

In summary, the mapped literature indicates active and growing investigation of GI POCUS for visualizing gastrointestinal dynamics at the ICU bedside. Translating these descriptive findings into clinical practice requires rigorous methodological consensus, multicenter pragmatic validation, structured training programs, and evaluation of safety and patient-important outcomes.

### 4.6 Limitations

#### 4.6.1 Limitations of the current evidence and clinical practice

Despite GI POCUS advancing beyond symptomatic assessment to visualize functional dynamics, three critical challenges persist. First, overfocus on isolated gastric evaluation causes fragmented physiological assessment, neglecting gastrointestinal coordination essential for digestion and compromising accurate ENI risk stratification. Second, heterogeneous acoustic parameters and non-standardized protocols lead to operational complexity and operator dependency. Third, the lack of unified, evidence-based protocols and standardized pathways across different phases of critical illness hinders systematic implementation and cross-institutional consistency.

#### 4.6.2 Methodological limitations of this scoping review

Several methodological limitations of this scoping review must be acknowledged.

First, no prospectively registered or publicly verifiable *a priori* protocol was available for this review. While the review was conducted in accordance with JBI scoping review methodology and a protocol was developed internally before study selection, the absence of external registration limits full transparency and reproducibility of the review process. No substantial deviations from the planned eligibility criteria occurred during the review; the PCC framework was applied uniformly at both screening stages.

Second, formal critical appraisal (risk of bias assessment) was not performed, consistent with established scoping review methodology. Consequently, the reported findings reflect what primary studies stated rather than an assessment of their methodological quality, and efficacy- or accuracy-oriented conclusions should be interpreted with appropriate caution. Similarly, no meta-analytic pooling was conducted due to substantial clinical and methodological heterogeneity; this review therefore maps the breadth and distribution of reported outcomes but cannot draw definitive conclusions regarding clinical effectiveness.

Third, geographic and language bias is present. The vast majority of included records originated from China, which reflects active regional exploration but limits international transportability; international multicenter trials, particularly those involving geographically and health-system diverse populations, are required to confirm global generalizability. Additionally, the review was restricted to English and Chinese publications because the review team could reliably assess full texts in both languages without external translation; this avoided misclassification that could arise from machine or third-party translation. Nevertheless, this restriction may have omitted relevant studies published in other languages, and international multicenter trials in geographically and health-system diverse populations are required to confirm global generalizability.

Fourth, the predominance of small, single-center studies with uncertain sponsorship patterns limits the generalizability of findings. While primary-study funding sources and COI declarations were systematically extracted, the absence of COI reporting in a majority of studies limits interpretation of potential sponsorship bias, and the overall evidence base remains dominated by preliminary, single-institution investigations.

Fifth, inter-rater concordance for study screening was not prespecified, and the raw pre-consensus screening decisions were not retained in a form that permits a valid retrospective calculation of a kappa statistic. In addition, the article-level linkage between individually excluded full-text reports and their assigned primary reasons was not retained after screening; individual exclusion decisions therefore cannot be independently audited, and only aggregate categorical counts are reported. To mitigate selection bias, both title/abstract and full-text screening were performed independently by two reviewers, a calibration exercise was conducted on a 10% subset before formal screening, and disagreements were resolved through discussion and third-reviewer adjudication. These procedures reduced initial disagreement but do not eliminate the risk of selection bias.

Finally, several technical acquisition and operator variables of interest (such as the anatomical scanning plane, the formula used to estimate gastric residual volume from cross-sectional area, device and transducer type, number of scans, blinding, and inter−/intra-observer reliability) were not reported, or were reported too inconsistently, in the primary studies to permit reliable and comparable extraction and synthesis.

### 4.7 Future perspectives and proposed management framework

To contextualize these evidence gaps and guide future prospective investigation, we synthesized the mapped literature into a preliminary, hypothesis-generating conceptual GI POCUS-informed enteral nutrition research framework (Figure 4). This author-developed framework summarizes concepts mapped in the review; it is not a validated clinical pathway, treatment algorithm, or recommendation for patient care, and its components and decision points require prospective feasibility, safety, and effectiveness evaluation.

Two components of this framework, however, extend beyond the mapped literature and warrant explicit justification. Ultrasonography-guided enteral feeding tube placement is supported by external evidence, including a recent meta-analysis and a scoping review of bedside ultrasound-guided placement (86, 87), with feasibility confirmed in mechanically ventilated and nurse-led settings (88, 89). The vascular component, linking splanchnic venous congestion and portal hemodynamics to gastrointestinal function and enteral nutrition, is supported by complementary studies in heart failure, liver transplantation, abdominal surgery, and hemodialysis (90–93). These data support the hypothesis that splanchnic congestion contributes to feeding intolerance, although direct ICU evidence and standardized sonographic quantification are lacking. Both components therefore remain hypothesis-generating extensions requiring prospective validation, which our group is currently undertaking.

## 5 Conclusion

This scoping review mapped 49 studies describing the use of GI POCUS for gastrointestinal assessment, prediction, and enteral-nutrition protocol adjustment in critically ill adults. The evidence was predominantly from China, focused mainly on gastric measures, and heterogeneous in populations, acquisition methods, operators, thresholds, and outcome definitions. Consistent with scoping-review methodology and the marked heterogeneity of the mapped literature, this descriptive evidence map does not establish clinical effectiveness, universal thresholds, or a validated management pathway. Future prospective multicenter studies should standardize definitions and acquisition protocols, report effect estimates with confidence intervals and patient-important outcomes, assess inter-operator reliability and training, and externally validate decision algorithms across diverse health systems.
