# Supplementary material for: Gastrointestinal point-of-care ultrasound for enteral nutrition management in critically ill adults: a scoping review
Source: Front Nutr. 2026 Oct 12;13:1972620. doi: 10.3389/fnut.2026.1972620 (PMC13696869; doi:10.3389/fnut.2026.1972620)
Supplement: Supplementary file 2 [file Table_1.DOCX]

Supplementary File 1: Detailed comprehensive search strategy

## PubMed

| **Concepts** | **Research Strategy Keywords** | **#** | **Results** |
| --- | --- | --- | --- |
| ICU  (Controlled Vocabulary) | "Critical Illness"[MeSH] OR "Catastrophic Illness"[MeSH] OR "Critical Care"[MeSH] | #1 | 111054 |
| ICU  (Free Text) | "critically ill patient"[Title/Abstract] OR "critical illness"[Title/Abstract] OR "serious illness"[Title/Abstract] OR "critical care "[Title/Abstract] OR "ICU"[Title/Abstract] OR "intensive care"[Title/Abstract] | #2 | 336963 |
| ICU | #1 OR #2 | #3 | 375887 |
| Enteral Nutrition  (Controlled Vocabulary) | "Enteral Nutrition"[MeSH] | #4 | 23896 |
| Enteral Nutrition  (Free Text) | "enteral nutrition"[Title/Abstract] OR "enteral feeding"[Title/Abstract] OR "tube feeding"[Title/Abstract] OR "nasogastric feeding"[Title/Abstract] | #5 | 23353 |
| Enteral Nutrition | #4 OR #5 | #6 | 34832 |
| Ultrasonography  (Controlled Vocabulary) | "Ultrasonography"[MeSH] | #7 | 529012 |
| Ultrasonography  (Free Text) | "ultrasonograph*"[Title/Abstract] OR "ultrasound*"[Title/Abstract] OR "sonograph*"[Title/Abstract] OR "POCUS"[Title/Abstract] OR "GIUS"[Title/Abstract] OR " Doppler*"[Title/Abstract] | #8 | 613773 |
| Ultrasonography | #7 OR #8 | #9 | 850495 |
| Total result | (#3 AND #6 AND #9) AND (("1900"[Date - Publication]: "2026/6/18"[Date - Publication])) | #10 | 188 |

## Embase

| **Concepts** | **Research Strategy Keywords** | **#** | **Results** |
| --- | --- | --- | --- |
| ICU  (Controlled Vocabulary) | ‘intensive care’/exp OR ‘critically ill patient’/exp | #1 | 1161760 |
| ICU  (Free Text) | ‘critically ill patient’:ti,ab,kw OR ‘critical illness’:ti,ab,kw OR ‘serious illness’:ti,ab,kw OR ‘critical care’:ti,ab,kw OR ‘ICU’:ti,ab,kw OR ‘intensive care’:ti,ab,kw | #2 | 564384 |
| ICU | #1 OR #2 | #3 | 1443213 |
| Enteral Nutrition  (Controlled Vocabulary) | ‘enteric feeding’/exp | #4 | 50094 |
| Enteral Nutrition  (Free Text) | ‘enteral nutrition’:ti,ab,kw OR ‘enteral feeding’:ti,ab,kw OR ‘tube feeding’:ti,ab,kw OR ‘nasogastric feeding’:ti,ab,kw | #5 | 39649 |
| Enteral Nutrition | #4 OR #5 | #6 | 61426 |
| Ultrasonography  (Controlled Vocabulary) | 'echography'/exp OR 'point of care ultrasound'/exp OR 'Doppler ultrasonography'/exp OR 'Doppler flowmetry'/exp | #7 | 1368726 |
| Ultrasonography  (Free Text) | ‘ultrasonograph*’:ti,ab,kw OR ‘ultrasound*’:ti,ab,kw OR ‘sonograph*’:ti,ab,kw OR ‘POCUS’:ti,ab,kw OR ‘GIUS’:ti,ab,kw OR ‘Doppler*’:ti,ab,kw | #8 | 949664 |
| Ultrasonography | #7 OR #8 | #9 | 1747969 |
| Total result | (#3 AND #6 AND #9) AND [<1966-2027]/py | #10 | 2746 |

Note: The year parameter up to 2027 was automatically generated by Embase to capture all "Article in Press" and "Early Access" records available at the time of search (June 18, 2026).

## Web of Science

| **Concepts** | **Research Strategy Keywords** | **#** | **Results** |
| --- | --- | --- | --- |
| ICU  (Free Text) | TS=("critically ill patient" OR "critical illness" OR "serious illness" OR "critical care " OR "ICU" OR "intensive care") | #1 | 371259 |
| Enteral Nutrition  (Free Text) | TS=("enteral nutrition" OR "enteral feeding" OR "tube feeding" OR "nasogastric feeding") | #2 | 26740 |
| Ultrasonography  (Free Text) | TS=("ultrasonography*" OR "ultrasound*" OR "sonograph*" OR "POCUS" OR "GIUS" OR "Doppler*") | #3 | 771154 |
| Total result | #1 AND #2 AND #3 AND PY= (1980-2026) | #4 | 174 |

## Cochrane Library

| **Concepts** | **Research Strategy Keywords** | **#** | **Results** |
| --- | --- | --- | --- |
| ICU  (Controlled Vocabulary) | #1 MeSH descriptor: [Critical Illness] explode all trees  #2 MeSH descriptor: [Catastrophic Illness] explode all trees  #3 MeSH descriptor: [Critical Care] explode all trees  #4 #1 OR #2 OR #3 | #4 | 6716 |
| ICU  (Free Text) | ("critically ill patient" OR "critical illness" OR "serious illness" OR "critical care " OR "ICU" OR "intensive care"):ti,ab,kw | #5 | 53024 |
| ICU | #4 OR #5 | #6 | 53040 |
| Enteral Nutrition  (Controlled Vocabulary) | MeSH descriptor: [Enteral Nutrition] explode all trees | #7 | 2610 |
| Enteral Nutrition  (Free Text) | ("enteral nutrition" OR "enteral feeding" OR "tube feeding" OR "nasogastric feeding"):ti,ab,kw | #8 | 7767 |
| Enteral Nutrition | #7 OR #8 | #9 | 7767 |
| Ultrasonography  (Controlled Vocabulary) | MeSH descriptor: [Ultrasonography] explode all trees | #10 | 20360 |
| Ultrasonography  (Free Text) | (ultrasonography* OR ultrasound* OR sonograph* OR POCUS OR GIUS OR Doppler*):ti,ab,kw | #11 | 78818 |
| Ultrasonography | #10 OR #11 | #12 | 83096 |
| Total result | #6 AND #9 AND #12 with Cochrane Library publication date up to June 18, 2026 | #13 | 102 |

## CINAHL

| **Concepts** | **Research Strategy Keywords** | **#** | **Results** |
| --- | --- | --- | --- |
| ICU  (Controlled Vocabulary) | (MH "Critical Illness+") OR (MH "Catastrophic Illness") OR (MH "Critical Care") | #S1 | 204994 |
| ICU  (Free Text) | "critically ill patient" OR "critical illness" OR "serious illness" OR "critical care " OR "ICU" OR "intensive care" | #S2 | 1568098 |
| ICU | #S1 OR #S2 | #S3 | 1572316 |
| Enteral Nutrition  (Controlled Vocabulary) | MH "Enteral Nutrition+" | #S4 | 36103 |
| Enteral Nutrition  (Free Text) | "enteral nutrition" OR "enteral feeding" OR "tube feeding" OR "nasogastric feeding" | #S5 | 73692 |
| Enteral Nutrition | #S4 OR #S5 | #S6 | 73692 |
| Ultrasonography  (Controlled Vocabulary) | MH "Ultrasonography+" | #S7 | 652157 |
| Ultrasonography  (Free Text) | "ultrasonograph*" OR "ultrasound*" OR "sonograph*" OR "POCUS" OR "GIUS" OR " Doppler*" | #S8 | 1596392 |
| Ultrasonography | #S7 OR #S8 | #S9 | 1754708 |
| Total result | #S3 AND #S6 AND #S9  Qualification - Publication date: 10000101-20260618 | #S10 | 492 |

## Scopus

| **Concepts** | **Research Strategy Keywords** | **#** | **Results** |
| --- | --- | --- | --- |
| ICU  (Free Text) | TITLE-ABS-KEY("critically ill patient" OR "critical illness" OR "serious illness" OR "critical care " OR "ICU" OR "intensive care") | #1 | 617986 |
| Enteral Nutrition  (Free Text) | TITLE-ABS-KEY("enteral nutrition" OR "enteral feeding" OR "tube feeding" OR "nasogastric feeding" ) | #2 | 42815 |
| Ultrasonography  (Free Text) | TITLE-ABS-KEY("ultrasonograph*" OR "ultrasound*" OR "sonograph*" OR "POCUS" OR "GIUS" OR " Doppler*") | #3 | 1221329 |
| Total result | (#1 AND #2 AND #3) AND PUBYEAR > 1995 AND PUBYEAR < 2027 | #4 | 309 |

## Chinese Databases

| **Concepts** | **Research Strategy Keywords** | **Results** |
| --- | --- | --- |
| China National Knowledge Infrastructure (CNKI) | (SU=急危重症 OR SU=危重症 OR SU=重症 OR SU=监护室 OR SU=ICU) AND (SU=肠内营养 OR SU=肠内喂养 OR SU=管饲 OR SU=鼻饲) AND (SU=超声 OR SU=B超)  发表时间：Inception to June 18, 2026  (Executed on June 18, 2026) | 143 |
| China Science and Technology Journal Database (VIP) | “任意字段=(急危重症+危重症+重症+监护室+ICU) AND 任意字段=(肠内营养+肠内喂养+管饲+鼻饲) AND 任意字段=(超声+B超)”，模糊匹配  发表时间：Inception to June 18, 2026  (Executed on June 18, 2026) | 312 |
| WanFang Data | (主题=急危重症 OR 主题=危重症 OR 主题=重症 OR 主题=监护室 OR 主题=ICU) AND (主题=肠内营养 OR 主题=肠内喂养 OR 主题=管饲 OR 主题=鼻饲) AND (主题=超声 OR 主题=B超)  发表时间：Inception to June 18, 2026  (Executed on June 18, 2026) | 386 |
| SinoMed (Chinese Biomedical Literature Service System) | ( "急危重症"[常用字段:智能] OR "危重症"[常用字段:智能] OR "重症"[常用字段:智能] OR "监护室"[常用字段:智能] OR "ICU"[常用字段:智能]) AND( "肠内营养"[常用字段:智能] OR "肠内喂养"[常用字段:智能] OR "管饲"[常用字段:智能] OR "鼻饲"[常用字段:智能]) AND( "超声"[常用字段:智能] OR "B超"[常用字段:智能]) AND Inception to June 18, 2026[日期]  (Executed on June 18, 2026) | 271 |

*Note on Database Search Date Ranges:

All electronic database searches across the 10 included platforms (English and Chinese) were executed on June 18, 2026. Where database interfaces default to open-ended time boundaries or include automated future calendar indexing for ahead-of-print / early access online records, the actual retrieval boundary strictly captured all literature published and indexed up to the final execution date of June 18, 2026.
