# Supplementary material for: Gastrointestinal point-of-care ultrasound for enteral nutrition management in critically ill adults: a scoping review
Source: Front Nutr. 2026 Oct 12;13:1972620. doi: 10.3389/fnut.2026.1972620 (PMC13696869; doi:10.3389/fnut.2026.1972620)
Supplement: Supplementary file 3 [file Table_3.DOCX]

Supplementary file 3: Definitions of gastrointestinal complications extracted from literature

| **Complications** | **Definition** | **Source** |
| --- | --- | --- |
| **Regurgitation** | Gastric contents reverse flow into the esophagus, pharynx, or oral cavity. | (Cao et al., 2017),  (Pan et al., 2019),  (Ren et al., 2019),  (Chen et al., 2019),  (Zheng et al., 2019),  (Pei and Shu, 2022),  (Jiang et al., 2022),  (Li et al., 2023),  (Sun and Cao, 2020),  (Zhang et al., 2022),  (He et al., 2022)  (Mao et al., 2025) |
|  | Occurrence of nutrient fluid in the mouth or overflow from the gastric tube at least once within 24 hours. | (Liu et al., 2024) |
|  | Presence of reflux detected via biochemical assay (e.g., detection of starch/maltodextrin from dairy products) in pharyngeal secretions sampled 4-8 hours after initiating EN. | (Zhang et al., 2011) |
|  |  |  |
| **Vomiting** | Nutrient solution appeared in the patient's mouth within 12 hours | (Zhao et al., 2021) |
|  | Stomach contents flowing out of the mouth and nose through the esophagus. | (Zhang et al., 2022),  (Xu et al., 2023) |
|  |  |  |
| **Aspiration** | Dyspnea, significant increase in pulmonary rales, sudden decrease in SpO₂, tachycardia, and aspiration of gastric contents from the airway. | (Cao et al., 2017),  (Zheng et al., 2019),  (Wang et al., 2021),  (Jiang et al., 2022),  (Pei and Shu, 2022),  (Liu et al., 2024),  (Mao et al., 2025) |
|  | Inhaled material enters the airway below the glottis. | (Pan et al., 2019),  (Chen et al., 2019),  (Li et al., 2023) |
|  | Gastric contents reflux into the pharynx and trachea due to gravity, increased intra-abdominal pressure, or elevated intragastric pressure. | (Ren et al., 2019), (Dong et al., 2022) |
|  | Gastric contents entering the airway or alveoli, visible via fiberoptic bronchoscopy or aspirated as obvious enteral nutrition fluid via suction catheter. | (Sun and Cao, 2020),  (He et al., 2022) |
|  |  |  |
| **Diarrhea** | Defecation frequency >3 times/day of unformed/liquid stools, often with other intestinal symptoms, or daily excretion of unformed/liquid stools >250 g. | (Pan et al., 2019) |
|  | Liquid/watery stools >3 times/day and stool volume >200-250 g/day or >250 mL/day. | (Ren et al., 2019),  (Fan et al., 2022),  (Li et al., 2023),  (Zhang et al., 2022),  (Li et al., 2024),  (Zou et al., 2019),  (Mao et al., 2025) |
|  | Defecation frequency ≥3 times/day, stool volume ≥250 mL/day or ≥200 g/day, with stool consistency corresponding to Types 5-7 on the Bristol Stool Form Scale. | (Chen et al., 2019),  (Huang et al., 2025) |
|  | Stool frequency >4 times/day, volume <500 mL/day, loose stool. | (Zhao et al., 2021) |
|  | Liquid/watery stools 3-5 times/day and total daily volume ≥500 mL. | (Dong et al., 2022) |
|  | Daily stool volume >500 mL with loose or watery consistency. | (He et al., 2022) |
|  | Daily defecations >3 times, feces thin/watery, high water content, daily stool volume >200 g. | (Xu et al., 2023) |
|  |  |  |
| **Gastric Retention** | Single aspiration of gastric residual >200 mL. | (Fan et al., 2022) |
|  | GRV ≥250 mL. | (Ni et al., 2024) |
|  | GRV >250 mL after 4 hours of continuous infusion. | (Zou et al., 2019),  (Yu et al., 2023) |
|  | GRV >250 mL after 4 hours of continuous infusion or GRV >500 mL within 24 hours. | (Li et al., 2024) |
|  | GRV >250 mL after 6 hours of continuous infusion | (Mao et al., 2025) |
|  | GRV >250 mL after 4 hours of continuous infusion or GRV >500 mL within 6 hours. | (Huang et al., 2025) |
|  | Nasogastric aspirate volume > 50% of the previously administered feed volume, or GRV > 400 mL | (Sujeesh et al., 2026) |
|  |  |  |
| **Abdominal Distension** | Intra-abdominal pressure (IAP) >12 mmHg measured twice consecutively within 6 hours. | (Ren et al., 2019),  (Li et al., 2023),  (Zhang et al., 2022) |
|  | Abdominal distension/tightness, tympany on percussion, radiographic signs of intestinal gas/fluid accumulation, and elevated IAP (IAP >12 mmHg measured twice consecutively within 6 hours). | (Fan et al., 2022) |
|  | Subjective feeling of abdominal bloating/fullness or IAP >15 mmHg. | (Zou et al., 2019),  (Li et al., 2024),  (Huang et al., 2025) |
|  | Discomfort caused by abdominal swelling or fullness. | (Xu et al., 2023) |
