# Supplementary material for: Gastrointestinal point-of-care ultrasound for enteral nutrition management in critically ill adults: a scoping review
Source: Front Nutr. 2026 Oct 12;13:1972620. doi: 10.3389/fnut.2026.1972620 (PMC13696869; doi:10.3389/fnut.2026.1972620)
Supplement: Supplementary file 4 [file Table_4.DOCX]

**Supplementary File 4. Comparison of the present scoping review with recent evidence syntheses on point-of-care ultrasound in enteral nutrition management**

| Characteristic | Jia et al.2026 [12] | Mihnovits et al.2024 [13] | Zhang et al.2026 [14] | Present review |
| --- | --- | --- | --- | --- |
| Design | Systematic review and meta-analysis | Narrative review with protocol proposal | Scoping review (Chinese-language) | Scoping review |
| Search end date | 12 Sep 2024 | Non-systematic; no stated end date | 4 Sep 2025 | 18 Jun 2026 |
| Databases and languages | 8 databases; English and Chinese | 3 databases; English | 9 databases; English and Chinese | 10 databases; English and Chinese |
| Population and context | Critically ill adults; diagnostic accuracy of GI ultrasound for feeding intolerance | Critically ill adults; gastrointestinal ultrasound | Critically ill adults; bedside ultrasound for GI function and EN guidance | Critically ill adults; GI POCUS to guide EN or predict EN intolerance and prognosis |
| Included studies | 16 studies; 1,261 participants | Non-systematic; not enumerated | 36 studies (19 RCTs, 15 case-control, 2 cohort) | 49 unique studies (36 interventional; 13 observational) |
| Anatomical domains covered | GI ultrasound predictors (predominantly gastric; composite scores) | Stomach and bowel (CSA, diameter, wall thickness, peristalsis) | Gastric (CSA, MI), intestinal, vascular (SMA), composite (AGIUS, GUTS) | Gastric (GRV, CSA, MI), intestinal (CD, CPF), vascular (SMA), composite (AGIUS) |
| Intervention protocols mapped | No | Partially (structural GIUS protocol proposed) | EN guidance mapped at assessment-scheme level; no algorithm synthesis | Yes (feeding-titration algorithms; GRV-guided four-zone framework) |
| Predictive accuracy assessed | Yes; pooled diagnostic accuracy (SROC-AUC) | No | Descriptive statements only;  no pooled estimates | Yes (38 AUC point estimates across 13 studies; descriptive map, no pooling) |
| Quality / risk-of-bias assessment | Yes (QUADAS-2) | No | Yes (NOS; RoB 2) | No (scoping review; explicitly stated) |
| Incremental contribution of the present review | Maps 36 interventional feeding-titration algorithms  Adds intestinal (CD, CPF) and vascular (SMA) parameters  Displays 38 descriptive AUC estimates  Visualizes outcome-reporting patterns  Search updated to June 2026 | Systematic evidence map of 49 studies  Synthesizes feeding-titration algorithms (GRV four-zone framework)  Displays 38 descriptive AUC estimates  Visualizes outcome-reporting patterns | English publication extending the Chinese-language map  Search updated to June 2026, international records  Synthesizes interventional feeding-titration algorithms  Displays 38 descriptive AUC estimates  Visualizes outcome-reporting patterns | — |

**Table legend.** AGIUS: Acute Gastrointestinal Injury Ultrasonography Score; AUC: area under the curve; CD: colon diameter; CPF: colonic peristaltic frequency; CSA: cross-sectional area; EN: enteral nutrition; GI: gastrointestinal; GI POCUS: gastrointestinal point-of-care ultrasound; GIUS: gastrointestinal ultrasound; GRV: gastric residual volume; GUTS: Gastrointestinal and Urinary Tract Sonography score; MI: motility index; NOS: Newcastle-Ottawa Scale; RCT: randomized controlled trial; RoB: risk of bias; SMA: superior mesenteric artery; SROC: summary receiver operating characteristic.
